# Primary allogeneic mitochondrial mix (PAMM) transfer/transplant by MitoCeption to address damage in PBMCs caused by ultraviolet radiation

**DOI:** 10.1186/s12896-019-0534-6

**Published:** 2019-06-28

**Authors:** Francisco Cabrera, Mayra Ortega, Francesca Velarde, Eliseo Parra, Stephany Gallardo, Diego Barba, Lina Soto, Gabriela Peña, Luis Alberto Pedroza, Christian Jorgensen, Maroun Khoury, Andrés Caicedo

**Affiliations:** 1 Colegio de Ciencias de la Salud, Escuela de Medicina Veterinaria, Universidad San Francisco de Quito, 17-12-841 Quito, Ecuador; 2 Colegio de Ciencias Biológicas y Ambientales, Escuela de Biotecnología, Universidad San Francisco de Quito, 17-12-841 Quito, Ecuador; 3 Colegio de Ciencias de la Salud, Escuela de Medicina, Universidad San Francisco de Quito, 17-12-841 Quito, Ecuador; 4 Baylor College of Medicine: Department of Pediatrics, Section of Immunology, Allergy, and Rheumatology, Baylor College of Medicine, Houston, TX USA; 5 IRMB CHU Saint Eloi, University of Montpellier, France, 80 rue Augustin Fliche, 34295 Montpellier, cedex 5 France; 6 Laboratory of Nano-Regenerative Medicine, Faculty of Medicine, Universidad de Los Andes, Santiago, Chile; 7 Consorcio Regenero, Chilean Consortium for Regenerative Medicine, Santiago, Chile; 8 Cells for Cells, Santiago, Chile; 9 Instituto de Investigaciones en Biomedicina, Universidad San Francisco de Quito, 17-12-841 Quito, Ecuador; 10 Mito-Act Research Consortium, Quito, Ecuador; 11 Sistemas Médicos, SIME, Universidad San Francisco de Quito, 17-12-841 Quito, Ecuador

**Keywords:** Mitochondria, MitoCeption, Artificial mitochondria transfer / transplant (AMTT), Primary allogeneic mitochondrial mix (PAMM), Ultraviolet radiation (UVR), Cellular damage, p53, Primary immune cells, Cell repair

## Abstract

**Background:**

Artificial Mitochondrial Transfer or Transplant (AMT/T) can be used to reduce the stress and loss of viability of damaged cells. In MitoCeption, a type of AMT/T, the isolated mitochondria and recipient cells are centrifuged together at 4 °C and then co-incubated at 37 °C in normal culture conditions, inducing the transfer. Ultraviolet radiation (UVR) can affect mitochondria and other cell structures, resulting in tissue stress, aging, and immunosuppression. AMT/T could be used to repair UVR cellular and mitochondrial damage. We studied if a mitochondrial mix from different donors (Primary Allogeneic Mitochondrial Mix, PAMM) can repair UVR damage and promote cell survival.

**Results:**

Using a simplified adaption of the MitoCeption protocol, we used peripheral blood mononuclear cells (PBMCs) as the recipient cell model of the PAMM in order to determine if this protocol could repair UVR damage. Our results showed that when PBMCs are exposed to UVR, there is a decrease in metabolic activity, mitochondrial mass, and mtDNA sequence stability as well as an increase in p53 expression and the percentage of dead cells. When PAMM MitoCeption was used on UVR-damaged cells, it successfully transferred mitochondria from different donors to distinct PBMCs populations and repaired the observed UVR damage.

**Conclusion:**

Our results represent an advancement in the applications of MitoCeption and other AMT/T. We showed that PBMCs could be used as a PAMM source of mitochondria. We also showed that these mitochondria can be transferred in a mix from different donors (PAMM) to UVR-damaged, non-adherent primary cells. Additionally, we decreased the duration of the MitoCeption protocol.

**Electronic supplementary material:**

The online version of this article (10.1186/s12896-019-0534-6) contains supplementary material, which is available to authorized users.

## Background

A substantial number of in vitro and in vivo assays have demonstrated the natural ability of cells to transfer mitochondria amongst each other [1]. This phenomenon is most commonly observed in mitochondrial transfer from healthy mesenchymal stem/stromal cells (MSCs) to damaged cells [2–7]. The transfer replaces or repairs damaged mitochondria and thereby reduces the percentage of dead cells and restores normal functions [3, 4, 8]. In 1982, Clark and Shay introduced a type of AMT/T model using a co-incubation step between the recipient cell and exogenous mitochondria [9]. Their pioneering study demonstrated for the first time that the mitochondrial DNA (mtDNA) of donor cells could be integrated into recipient cells and subsequently transmit hereditary traits and induce functional changes. AMT/T mimics the natural process of mitochondrial transfer, reprograms cellular metabolism, and induces proliferation [10–13]. The introduction of this model elucidated the possible use of mitochondria as an active therapeutic agent.

Since 1982, numerous adaptations of AMT/T have been developed for in vitro and in vivo applications [10–12]. Among all available methods, the use of a centrifugation during co-incubation seems to reduce the quantity of mitochondria needed to facilitate successful mitochondrial internalization by the recipient cells [11, 14, 15]. In-vitro cultured cells, especially MSCs, have been used as one of the most common sources of mitochondria for AMT/T [11, 12, 14]. However, using stem cells or other cultured cells, which require an extensive time to proliferate, increases the cost and reduces time-effectiveness of the process. Furthermore, a large number of cells are needed to successfully obtain high yields of mitochondria for transfer. As an advancement in AMT/T, McCully et al. successfully transplanted autologous mitochondria from skeletal muscle and injected them into damaged myocardium after ischemic injury, which lead to an improvement in ventricular function in humans [16].

Our study tests a modification of the original MitoCeption protocol which reduces the time and complexity of the protocol. We sought to determine if primary allogenic mitochondrial mix (PAMM) MitoCeption could be used to repair peripheral blood mononuclear cells (PBMCs) damaged by ultraviolet radiation (UVR) (UVC-UVR wavelength of 254 nm). PAMM is composed of the PBMCs of at least three donors. A secondary objective was to provide further evidence as to how UVR affects mitochondria and cell viability. To first determine the effects of UVR on cells and mitochondria, we created a cellular model in which human PBMCs were irradiated with UVR. Mitochondrial damage was assessed according to changes in mitochondrial mass, metabolic activity estimated by the 3-(4,5-Dimethylthiazol-2-yl)-2,5-diphenyltetrazolium bromide (MTT) assay, and percentage of dead cells; these indicators were examined 30 min to 120 min after (early time point) and 18 h after (late time point) exposure to radiation. Then, we selected a standard exposure time of 3 min for the protocol, because this level of UVR exposure resulted in harm but not complete cell death. Irradiated cells were rescued with varying doses of mitochondria isolated from different PBMC donors (PAMM) using the updated MitoCeption protocol. Using this approach, we showed that PAMM transfer from PBMC donors can repair UVR damage in recipient PBMCs. PBMCs can internalize PAMM and decrease the percentage of dead cells together with the repairing effect of immune cells’ respiratory burst (RB) after 18 h. Here, we describe a new method to repair UVR-damaged mitochondria using PAMM MitoCeption; 1 h to 2 h after PAMM MitoCeption, we determined that exogenous mitochondria had been internalized by recipient cells and normal cell viability and mitochondrial mass, metabolic function, DNA patrimony, p53 expression, and RB had been recuperated.

## Results

### UVR exposure decreases PBMC mitochondrial mass and metabolic function and increases the percentage of dead cells

To understand the effects of UVR on cell physiology, we developed an in vitro model that used fresh PBMCs and exposed them to a gradient of increasing doses of UVR during different lengths of time. For these assays, we isolated PBMCs from healthy donors of different ages and genders. PBMCs were exposed to increasing doses of UVC from 2 min (48 mJ/cm^2^) to 6 min (144 mJ/cm^2^). The effects of UVR on mitochondrial mass, function, and percentage of dead cells were measured at two separate times: immediately after exposure (early time point) and 18 h after culture (late time point). To test the effects of UVR on mitochondrial mass, we labeled the mitochondria with MitoTracker Green® and measured changes using flow cytometry with mean fluorescence intensity values (MFI). The shape, size, and granularity of treated and untreated PBMCs were analyzed (Additional file 1: Figure S1). The metabolic activity of PBMCs was measured with spectrophotometry using the MTT assay. Mitochondrial mass data was analyzed using flow cytometry and MTT assay for metabolic activity and subsequently normalized using sample/control-average transformation [17]. Finally, we stained PBMCs with Trypan Blue to determine the percentage of dead cells.

**Fig. 1:**
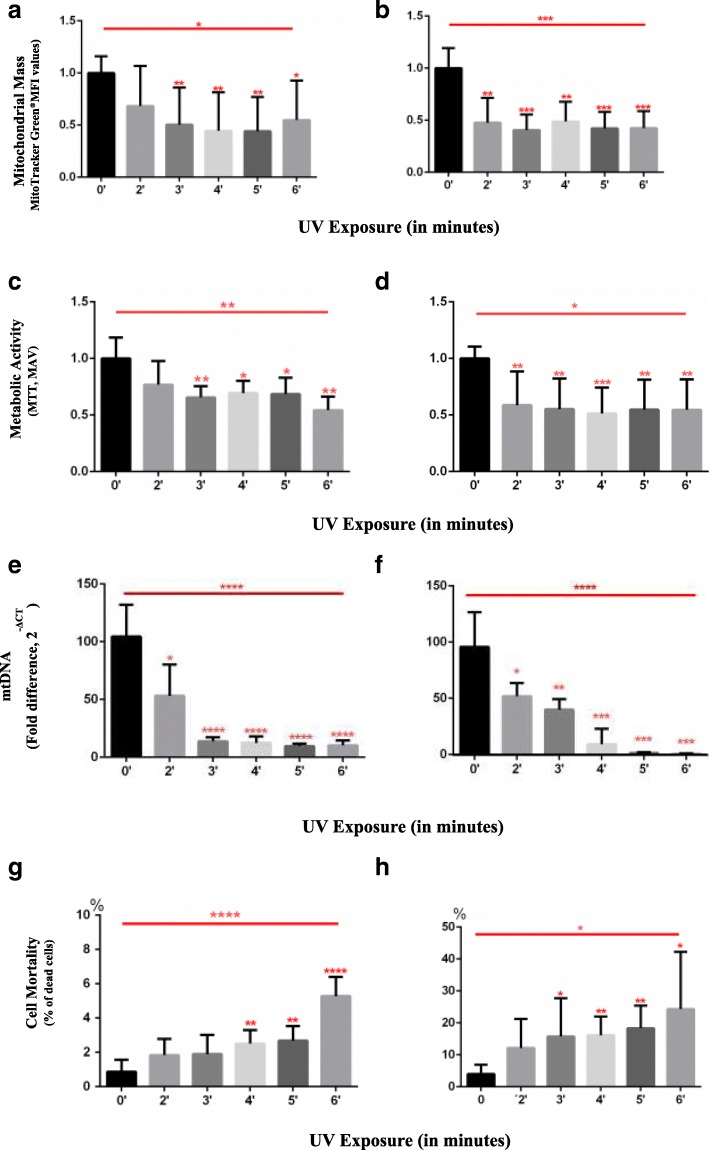
PBMCs’ mitochondrial mass, metabolic activity, mortality, and mtDNA quantification after UVR exposure. **a**, **b** Mitochondria mass (*n* = 8, 8 PBMC donors) determined by the mean fluorescence intensity values (MFI) of mitochondria labelled with MitoTracker® Green. **a** 1 h culture after exposure and 30’of MitoTracker® Green incubation. **b** 18 h culture and 30’of MitoTracker® Green incubation. Analysis, the sample/control-average transformation was used, Anderson-Darling normality test was applied. Un-paired, ANOVA test and Student’s t-test are shown in the figure with an alpha-value of 0.05 (*p < 0.05, **p < 0.01, ***p < 0.001, ****p < 0.0001) to observe statistically significant differences. **c**, **d** Metabolic activity measured by MTT (*n* = 5, 5 PBMC donors). Mean absorbance values (MAV) were measured by spectrophotometry of PBMCs treated with MTT after UVR exposure. **c** After 2 h incubation with MTT. **d** After 18 h in culture and 2 h incubation with MTT. Analysis: the sample/control-average transformation was used, Anderson-Darling normality test was applied. Un-paired, ANOVA test and Student’s t-test are shown in the figure with an alpha-value of 0.05 (*p < 0.05, **p < 0.01, ***p < 0.001, ****p < 0.0001) to observe statistically significant differences. **e**, **f** Determination of the effects on the mtDNA sequence of PBMCs after the exposure to UVR by qPCR (n = 8, 8 PBMC donors). The qPCR 2^-ΔCT^ fold method was used. Primers: HB2M as the housekeeping gene sequence (Human B2M Beta-2-microglobulin, NCBI AH002619.1.), and HMito (designed for the mitochondrial genome, between positions 241 and 390, NCBI NC_012920.1) from Ajaz et al.(2015) [18] **e** 1 h culture after exposure. **f** After 18 h culture. Analysis: Un-paired, ANOVA test and Student’s t-test are shown in the figure with an alpha-value of 0.05 (*p < 0.05, **p < 0.01, ***p < 0.001, ****p < 0.0001) to observe statistically significant differences. **g**, **h** Mortality of PBMCs induced by UVR (*n* = 7, 7 PBMC donors). The percentage of dead cells was estimated by counting those positive for Trypan Blue staining and then dividing them by the total; non-labelled viable cells were considered as well. **g** 1 h culture after exposure. **h** After 18 h culture. Statistical analysis: Un-paired, ANOVA test and Student’s t-test are shown in the figure with an alpha-value of 0.05 (*p < 0.05, **p < 0.01, ***p < 0.001, ****p < 0.0001) to observe statistically significant differences

We observed a statistically significant reduction (ANOVA test, *p < 0.05) in the mitochondrial mass of the lymphocytes (PBMCs) immediately after UVR exposure especially from 3 min (72 mJ/cm^2^) to 6 min (144 mJ/cm^2^) (Fig. 1a). Other specific PBMC populations differed in their mitochondrial masses and varied responses to UVR damage were observed (data not shown). After 18 h of culture, lymphocytes in the PBMCs that survived the UVR exposure did not recover their mitochondrial mass in most of the conditions tested (ANOVA test, ***p < 0.001) (Fig. 1b). The MTT assay showed a reduction of metabolic activity of the PBMCs in all conditions tested when exposed to increasing UVR exposure of (ANOVA test, **p < 0.01) (Fig. 1c). After 18 h of culture, viable cells still exhibited reduced metabolic activity (ANOVA test, *p < 0.05) (Fig. 1d). mtDNA damage data was analyzed using the qPCR 2^-ΔCT^ fold method; the level of damage was estimated by hMito primers’ failure to recognize the mtDNA sequence using qPCR with a nuclear housekeeping gene (hB2M). hMito primers bind to a unique region of the mtDNA (between positions 241 and 390), which has been shown to be less similar to nuclear DNA sequences, thereby ensuring more accurate identification by the primers [18, 19]. hB2M is the nuclear human β-2 microglobulin gene [18, 19]. We observed a statistically significant and proportional reduction in the number of copies of mtDNA after all UVR dosages at both time measurements (ANOVA test, ****p < 0.0001) (Fig. 1e, f). Following UVR exposure, measurement at the early time point revealed that 10% of the total number of cells were dead (Fig. 1g). After 18 h of culture, PBMCs showed a statistically significant (ANOVA test, *p < 0.05) increase in the percentage of dead cells following exposure to a higher dose of UVR (Fig. 1h).

### MitoCeption protocol update to facilitate the AMT/T using PAMM donors, unattached recipient cells, and alternative cell support in less time

In MitoCeption, the isolated mitochondria and recipient cells are centrifuged together at 4 °C and then co-incubated at 37 °C at normal culture conditions, inducing the transfer. The centrifugation and thermic shock are key steps in the protocol which successfully transfer small quantities of mitochondria to recipient cells [11]. The protocol was modified and adapted to transfer mitochondria to unattached PBMCs without using a plate or coating of a well surface as cell support. The cell support was changed from plates to 1.5 mL micro-centrifuge tubes (Fig. 2.1). We reduced centrifugation force (500 x g for 5 min at 4 °C) and exposure time to exogenous mitochondria (1 h); statistically significant (ANOVA test, ***p < 0.001) exogenous mitochondria up-take was still observed at 1 h and at 18 h (Fig. 2.2; 2.3).

**Fig. 2:**
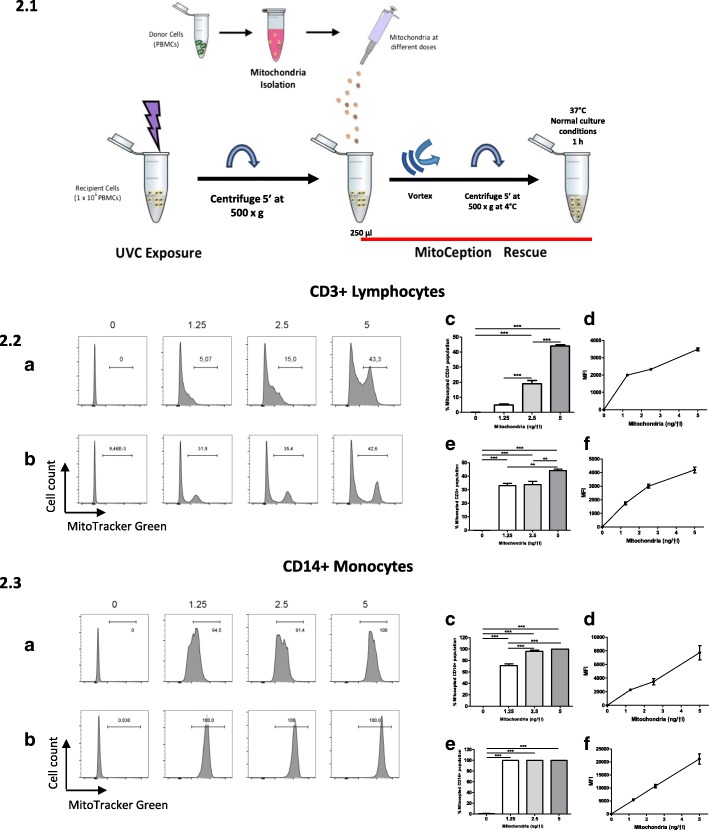
MitoCeption protocol update. (1) Schema of the updated protocol. Unattached recipient cells can be MitoCepted in a microcentrifuge tube of 1.5 ml and then centrifuged at 500 x g at during 5 min and immediately put in incubation for at least 1 h with mitochondria. Then, cells can be washed of excess mitochondria and used for downstream applications. Graphs show the proportional increase of the transfer of fluorescent mitochondria in percentages and MFIs due to the uptake in relation to the control of non-MitoCepted mitochondria. PAMM is composed of the PBMCs of at least three donors. (2, 3) MitoCepted CD3+ Lymphocytes and CD14+ Monocytes (*n* = 3, 3 PBMC donors, 3 PBMC donors for PAMM). Fresh PBMCs were MitoCepted with isolated mitochondria labeled with MitoTracker® Green from PBMCs; lymphocytes and monocytes were selected by their size, granularity, singles, alive cells (− for Annexin and 7AAD), and CD3+ and CD14+ identification. (2.2a, 2.2c, 2.2d) Flow cytometry of CD3+ cells after 1 h of MitoCeption. (2.2b, 2.2e, 2.2f) CD3+ after wash of excess mitochondria and 18 h of MitoCeption. (2.2c) CD3+ percentage of MitoCeption cells after 1 h. (2.2d) CD3+ MFI of the MitoCepted cells after 1 h. (2.2e) CD3+ Percentage of MitoCeption cells after 18 h. (2.2f) CD3+ MFI of the MitoCepted cells after 18 h. (2.3a, 2.3c, 2.3d) Flow cytometry of CD14+ cells after 1 h of MitoCeption. (2.3b, 2.3e, 2.3f) Flow cytometry of CD14+ cells after 18 h of MitoCeption. (2.3c) CD14+ percentage of MitoCeption cells after 1 h. (2.3d) CD14+ MFI of the MitoCeption cells after 1 h. (2.3e) CD14+ Percentage of MitoCeption cells after 18 h. (2.3f) CD14+ MFI of the MitoCepted cells after 18 h. Statistical analysis for all conditions: Mean ± SEM one-way ANOVA and Tuckey’s pot-test. (*p < 0.05, **p < 0.01, ***p < 0.001)

Using flow cytometry, we observed that the patterns of exogenous mitochondria internalization by recipient PBMCs subjected to the MitoCeption protocol varied according to cell type and population (Fig. 2.2; 2.3; Additional file 1: Figure S1). In CD3+ lymphocytes, two uptake patterns were observed. In one population, cells incorporated between 5 and 40% of exogenous mitochondria in proportion to the increasing dose of PAMM. Another population of CD3+ lymphocytes did not internalize mitochondria (Fig. 2.2.a, 2.2c-d). In contrast, after one hour, 60 to 100% of CD14+ monocytes had successfully internalized exogenous mitochondria from the lowest (1.25 ng/μl) to the highest (5 ng/μl) mitochondrial concentration; the mitochondrial uptake rate in CD14+ monocytes was proportional to increasing exogenous mitochondrial doses, although the observed effect was not as substantial as in the case of CD3+ lymphocytes (Fig. 2.3.a, 2.3c-d). In both CD3+ lymphocytes and CD14+ monocytes, a better definition of the fluorescence peak and a stabilization of the population that internalized the exogenous mitochondria was observed independently of the concentration, 30 to 40% for the lymphocytes and 100% for the monocytes (Fig. 2.2.b − 2.2.e-f; 2.3.b-2.3.e-f). In both CD3 + lymphocytes and CD14+ monocytes recipient cells, we observed an increase in fluorescence and MFI proportional to the concentration of mitochondria and successful mitochondria internalization after 1 h and 18 h (Fig. 2.2a-b; 2.2.d; 2.2.f; 2.3a-b; 2.3.d; 2.3.f).

### PAMM transfer of mitochondria by MitoCeption rescues mitochondrial mass, function, and viability in PBMCs damaged by UVR

**Fig. 3:**
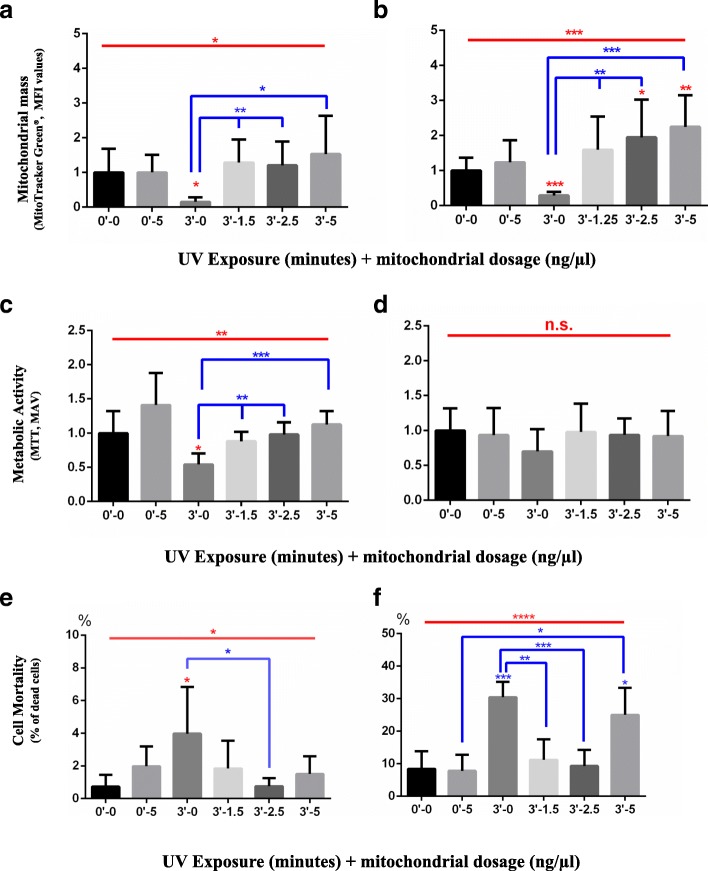
Estimation of the rescue of mitochondrial mass, metabolic activity, and mortality of damaged PBMCs using PAMM MitoCeption. PBMCs were isolated, exposed to UVR (3 min = 72 mJ/cm^2^), and treated with 3 allogeneic mitochondria doses (1,25-2,5–5,0 μg of mitochondria). **a**, **b** Mitochondria mass (n = 7, 7 PBMC donors exposed to UVR and PAMM of 3 donors). MFI of PBMCs labelled with MitoTracker Green® was analyzed by flow cytometry. **a** 1 h after exposure to UVR, 1 h after MitoCeption, wash, and 30` incubation with MitoTracker® Green. **b** After 1 h exposure to UVR, 1 h MitoCeption, wash and 18 h in culture. Analysis, the sample/control-average transformation was used, Anderson-Darling normality test was applied. Un-paired, ANOVA test and Student’s t-test are shown in the figure with an alpha-value of 0.05 (*p < 0.05, **p < 0.01, ***p < 0.001, ****p < 0.0001) to observe statistically significant differences. **c**, **d** Metabolic activity measured by MTT (*n* = 5, 5 PBMC donors exposed to UVR and PAMM of 3 donors). MAV were measured by spectrophotometry of PBMCs treated with MTT after UVR exposure and MitoCeption at the early and late time points. **c** 1 h after exposure to UVR, 1 h of MitoCeption, wash and 2 h of incubation with MTT. **d** 1 h after exposure to UVR, 1 h of MitoCeption, wash, 18 h in culture and 2 h of incubation with MTT. Analysis: the sample/control-average transformation was used, Anderson-Darling normality test was applied. Un-paired, ANOVA test and Student’s t-test are shown in the figure with an alpha-value of 0.05 (*p < 0.05, **p < 0.01, ***p < 0.001, ****p < 0.0001) to observe statistically significant differences. **e**, **f** Mortality of PBMCs caused by UVR (n = 5, 5 PBMC donors exposed to UVR and PAMM of 3 donors), the percentage was estimated by counting dead cells positive for Trypan Blue staining and dividing that number by viable cells. **e** Early time point measured 1 h after exposure to UVR, 1 h MitoCeption and wash **f** Late time point 1 h after exposure to UVR, 1 h MitoCeption, wash and 18 h in culture. Analysis: Anderson-Darling normality test was applied to the resulting data. Un-paired, ANOVA test and Student’s t-test are shown in the figure with an alpha-value of 0.05 (*p < 0.05, **p < 0.01, ***p < 0.001, ****p < 0.0001) to observe statistically significant differences

We demonstrated that UVR exposure in PBMCs cause a reduction of mitochondrial mass and function, loss of or changes in mtDNA, and an increase in the percentage of dead cells (Fig. 1a-h). Among all UVR doses tested, we determined that 3 min (72 mJ/cm^2^) was the minimal dose required to induce statistically significant cell and mitochondrial damage (ANOVA test, **p < 0.01); we used this UVR dose in all of the tested conditions. PBMCs damaged by 3 min of UVR exposure (72 mJ/cm^2^) were MitoCepted during 1 h with allogeneic mitochondria from different PBMCs donors (PAMM) and subsequently washed to remove excess exogenous organelles that were not successfully internalized (Fig. 3). Isolated mitochondria were MitoCepted in doses of 1.25, 2.5, and 5 ng/μl to 1 × 10^6^ UVR-damaged PBMCs.

Additionally, we used 5 ng/μl of isolated mitochondria to MitoCept PBMCs that had not been exposed to UVR in order to observe cytotoxicity. As expected, controls irradiated with a 3 min dose of UVR (72 mJ/cm^2^) exhibited a statistically significant decrease in their mitochondrial mass and function and increase in percentage of dead cells (Fig. 3a-f). Treatment with PAMM induced a complete recovery of mitochondrial mass and function and reduced percentage of dead cells at the early and late time points in the lymphocytes population (PBMCs) (Fig. 3a-f). In both the non-exposed PBMCs and those exposed to 3 min (72 mJ/cm^2^) of UVR which were then MitoCepted with 5 ng/μl of mitochondria, an increase of dead cells and mitochondrial mass was observed at the early time point; mitochondrial mass increase was more substantial in the UVR-damaged PBMCs. Additionally, this finding may indicate that internalization of excessive of mitochondria could be harmful to the cell (Fig. 3e). We did not observe adverse effects associated with the use of allogeneic mitochondria in doses ranging from 1.25 to 2.5 ng; there was no increase in the percentage of dead cells associated with these dosages (Fig. 3e-f). Additionally, 1 h exposure to exogenous mitochondria was enough to recover the loss of mitochondrial mass, metabolic activity, and percentage of dead cells in UVR-damaged recipient cells.

### Rescue of the loss of mtDNA hMito sequence and normalization p53 mRNA expression in UVR-damaged PBMCs using PAMM MitoCeption

**Fig. 4:**
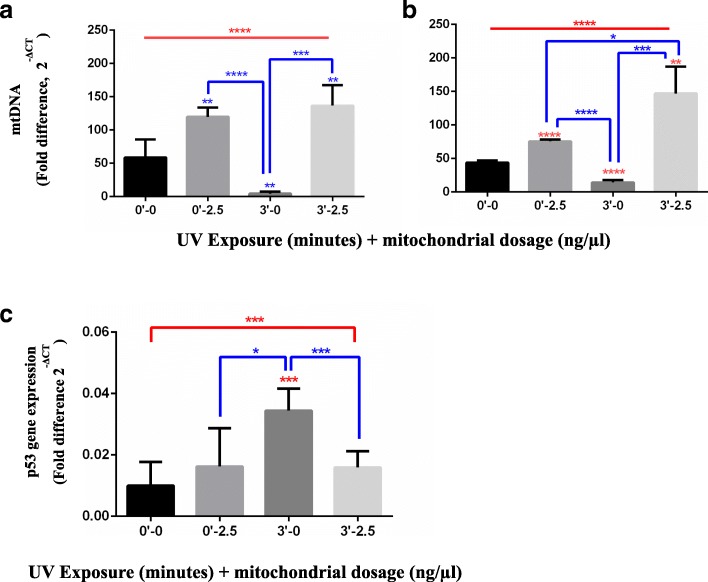
Determination of the effects on the mtDNA sequence, changes in the expression of the p53 gene induced by UVR, and rescue by adding exogenous mitochondria using PAMM MitoCeption in the PBMC model. **a**, **b** mtDNA damage quantification by the 2^-ΔCT^ fold by qPCR after 3 min UVR exposure and rescue by PAMM Mitoception (n = 5, 5 PBMC donors exposed to UVR and PAMM of 3 donors). The qPCR 2^-ΔCT^ fold method was used. Primers: HB2M as the housekeeping gene sequence (Human B2M Beta-2-microglobulin, NCBI AH002619.1.), and HMito (designed for the mitochondrial genome, between positions 241 and 390, NCBI NC_012920.1) from Ajaz et al.(2015) [18] **a** 1 h culture after UVR exposure and PAMM MitoCeption. **b** 18 h culture after UVR exposure and PAMM MitoCeption. Analysis: Un-paired, ANOVA test and Student’s t-test are shown in the figure with an alpha-value of 0.05 (*p < 0.05, **p < 0.01, ***p < 0.001, ****p < 0.0001) to establish statistically significant differences. **c** Analysis of the p53 gene expression after UVR damage and PAMM MitoCeption (*n* = 6, 6 PBMC donors exposed to UVR and PAMM of 3 donors). Primers for detecting p53 mRNA and for the nuclear PUM (housekeeping) were used to estimate p53 levels after UVR exposure and PAM MitoCeption in receptor cells with or without UVR damage. p53 gene expression was measured by qRT-PCR at 2 h after exposure and subsequent MitoCeption. Statistical analysis: Un-paired, ANOVA test and Student’s t-test are shown in the figure with an alpha-value of 0.05 (*p < 0.05, **p < 0.01, ***p < 0.001, ****p < 0.0001) to establish statistically significant differences. Un-paired, ANOVA test and Student’s t-test (p < 0.05)

After determining the appropriate UVR dose (3 min) in our PBMC model and the quantity of exogenous mitochondria (2.5 ng / μl by 1 × 10^6^ cells) needed to achieve statistically significant improvements in damaged cells, we assessed if the UVR damage of mtDNA could induce changes in p53 gene expression and if this could be repaired by PAMM MitoCeption. UVR caused a statistically significant loss of the mtDNA recognition sequence for hMito primers by qPCR, which was reversed by PAMM MitoCeption at both the early and late time points (Fig. 4a-b). UVR exposure caused a statistically significant increase (ANOVA test, ***p < 0.001) in p53 gene expression, as measured by qRT-PCR at 2 h after exposure and subsequent MitoCeption; this increase was then reversed by MitoCeption (Fig. 4c). We chose to measure the mRNA of p53 at 2 h because in preliminary assays the p53 expression at 1 h or after 18 h was minimally detected.

### Rescue of RB (ROS production) of damaged PBMCs by MitoCeption

**Fig. 5:**
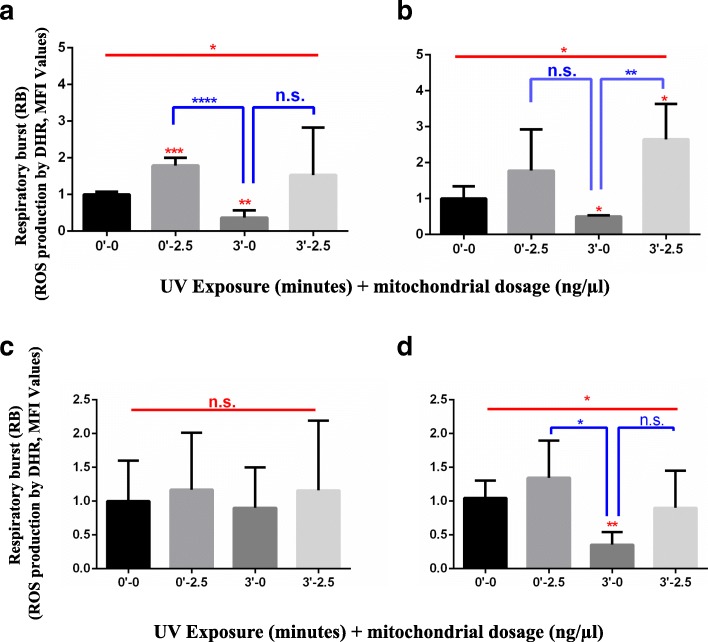
Estimation of the RB (ROS production) of UVR-exposed PBMCs (monocytes and lymphocytes) and assessment of PBMC rescue by PAMM MitoCeption. **a**, **b**, **c**, **d** ROS production measured with DHR-PMA treatment and flow cytometry (n = 6, 6 PBMC donors exposed to UVR and PAMM of 3 donors) **a**. Monocytes behavior 1 h after 3 min exposure to UVR and rescue by PAMM MitoCeption. **b**. Monocytes behavior after 18 h culture and rescue by PAMM MitoCeption. **c**. Lymphocytes behavior 1 h after 3 min exposure to UVR and rescue by PAMM MitoCeption. **d**. Lymphocytes behavior after 18 h culture and rescue by PAMM MitoCeption. Analysis: the sample/control-average transformation was used, Anderson-Darling normality test was applied. Un-paired, ANOVA test and Student’s t-test are shown in the figure with an alpha-value of 0.05 (*p < 0.05, **p < 0.01, ***p < 0.001, ****p < 0.0001) to observe statistically significant differences

We used flow cytometry analysis of lymphocyte and monocyte size and granularity (Additional file 1:Figure S1) in order to determine the level of ROS production by Phorbol 12-myristate 13-acetate (PMA); we detected ROS production using Dihydro-rhodamine-123 (DHR-123) in all conditions tested. PBMCs monocytes responded to PMA by increasing ROS production (also known as RB) in all tested conditions (Fig. 5a-b). Monocytes that were MitoCepted increased their ROS production quantity at the early and late time points. After the UVR damage, monocytes decreased their ROS production and recovered it after MitoCeption; the statistical significance of these results was confirmed using ANOVA and Student’s t-test (ANOVA test, *p < 0.05). The lymphocytic population did not exhibit statistically significant change in ROS production at the early time point. However, after 18 h, the lymphocyte population exposed to 3 min of UVR damage showed a statistically significant reduction (ANOVA test, **p < 0.01) in ROS production (Fig. 5c-d). After MitoCeption, PMA-stimulated lymphocytes showed a slight increase in ROS production (Fig. 5a-b). After 18 h, the UVR-damaged lymphocytes partially recovered their ROS production following MitoCeption.

## Discussion

MitoCeption has been shown to reprogram cell metabolism and increase cancer cell proliferation and invasion 24 h after the transfer of mitochondria isolated from MSCs [11]. Our results showed that MitoCeption rescues the loss of mitochondrial mass, function, and viability as soon as 1 h after cells were exposed to UVR. Here, we adapted the MitoCeption protocol to be used with fresh non-adherent immune cells such as PBMCs and changed the exogenous mitochondria source, centrifugation time and force, co-incubation time, and cell support. Some protocols use MSCs as a mitochondria source; however, the reduced proliferation capacity of MSCs makes obtaining sufficient cells impractical and expensive [14, 20, 21]. Therefore, we tested the suitability of PBMCs as the mitochondria source, which constitutes a novel approach in the field and is a more accessible mitochondria source because a greater number of mitochondria donor cells can be obtained from multiple donors. We decreased the force and time of centrifugation (500 x g for 5 min at 4 °C) and changed the original protocol’s volume of medium from 1 mL to 250 μL in order to maximize recipient cell and mitochondria interaction. Finally, we used a different supporting material by changing the culture plate to 1.5 mL microcentrifuge tubes, thereby adapting this protocol for use with unattached cells like PBMCs.

The MitoCeption protocol uses centrifugation to put recipient cells in close contact with the exogenous mitochondria and a thermic shock to attempt to facilitate mitochondrial uptake. It has been reported that MitoCeption requires 1.25 to 20 μg/ml of mitochondria (concentration measured using Bradford protein quantification assay) per 100,000 cancer cells to achieve phenotypical changes in the recipient cells [11]. MitoCeption allows minimal quantities of mitochondria to be used. The use of additional steps in co-incubation may depend on the user needs, cell type, and application. Studies performed by Lorberboum-Galski and her team showed that simple incubation between receiver cells and exogenous mitochondria was sufficient to facilitate transfer in a process called “Mitochondrial Transformation”. In most assays, her team used 300 μg / mL of mitochondria with 50,000 HepG2 cells, MCF-7, 30,000 HEK-293, or 75,000 fibroblasts, did not use centrifugation, and completed data collection 1 h and 24 h after combining the recipient cells and exogenous motochondria [15]. In our study, we found that we were able to obtain yields of 50 μg / mL of mitochondria using only 16 × 10^6^ PBMCs (4 donors), which was sufficient for all conditions tested. Interestingly, other groups have also found that performing a centrifugation step prior to the co-incubation of the isolated mitochondria with the recipient cells greatly improves the mitochondria uptake; for example, Mi Jin Kim et al. found that 0.05, 0.5 and 5 μg of exogenous mitochondria were successfully transferred to 10,000 recipient cells after 24 h [14].

To our knowledge, this study is the first to demonstrate in-vitro that MitoCeption can be used to re-establish mitochondrial function loss caused by UVR exposure. Additionally, we successfully transferred a mix of different PBMC donors to one PAMM that was used to repair damaged cells. Other research groups have successfully transferred mitochondria from one cell donor type to others [10, 11, 20]; however, none of them have mixed mitochondria isolated from different donors for the transfer/transplant. This study elucidates the potential to use mitochondria from different donors (PAMM) to treat UVR stress and possibly other types of damage or metabolic malfunctions in cells, resulting in not only in-vitro but also ex-vivo applications. The repairing capacity of the PAMM and MitoCeption protocols require further investigation because the effects beyond 18 h of culture, the long-term effects of multiple mitochondria patrimonies inside the recipient cells, and the capacity to re-use them in-vivo for distinct applications is unknown.

In our study, assessment of mitochondrial state in the PAMM was limited, although the PAMM demonstrated reparative properties. Our results demonstrate that PAMM MitoCeption can repair some cell damage inflicted by UVR exposure. In order to promote mitochondrial stability during co-culture, in our protocol, we reduced the centrifugation and co-incubation time and the possible cellular stress induced by the long centrifugation and thermic shock included in the original MitoCeption methodology. Nonetheless, it is crucial that further assays be performed to determine if the mitochondria in PAMM are active, energetically coupled or uncoupled, and structurally complete in order to fully understand their repairing properties.

We observed that UVR-exposed PBMCs exhibited a decrease in mitochondrial mass and metabolic activity as measured by MTT. Likewise, UVR exposure induced changes or loss of mtDNA and increased the percentage of dead cells. Furthermore, increased p53 gene expression was associated with changes or loss in the mtDNA target sequence of hMito primers. UVR exposure also caused a decrease in ROS production (especially in monocytes), thus affecting the RB. The RB is part of the innate response to infection and host defense based on ROS production by the NADPH oxidase enzyme, which is situated in the membranes of monocytes and which may be sensitive to UVR [22]. We provided further insight into how mitochondria are affected by UVR in PBMCs in order to validate the use of MitoCeption to rescue cells exposed to UVR-induced stress (Additional file 2: Figure S2).

UVC was used to generate a standard damaging procedure for PBMCs to validate the use of the MitoCeption of PAMM to repair this damage. UVC greatly disturbs DNA and particularly mtDNA; damage especially accumulates in the latter molecule due to the lack of mtDNA repairing systems as compared to the nucleus [23]. Moreover, when mtDNA reaches a threshold limit for damage, it causes mitochondrial malfunction, increased ROS production, and induces apoptosis [23–26]. By using UVC, our study showed that increasing doses of radiation causes proportional loss of mtDNA, mitochondrial mass integrity, and metabolic function as soon as 1 h after exposure.

Measurement of the mitochondrial mass by MitoTracker Green® has been proven to be an accurate method to distinguish differences among cells and experimental conditions [27, 28]. Once we successfully used MitoCeption to transfer isolated mitochondria to undamaged PBMCs and UVR-damaged PBMCs, flow cytometry was used to demonstrate that recipient cells exhibited an increase in mitochondrial mass proportional to the dose of transferred mitochondrial. Thus, a statistically significant increase in mitochondrial mass was observed primarily in recipient cells that had been co-cultured with exogenous mitochondria in concentrations greater than or equal to 2.5 ng / μl. After UVR damage, the decrease in percentage of dead cells was completely recovered by the adapted PAMM MitoCeption protocol.

In the MTT technique, the metabolic activity of living cells transforms the MTT chemical compound into formazan crystals, which appear blueish to violet in color. Once living cells have transformed the MTT in formazan crystals, dimethyl sulfoxide (DMSO) lysates the cells and dilutes the crystals. The viability and metabolic activity of the cells can be estimated by analyzing this bluish to violet solution with spectrophotometry [29]. The Trypan Blue dye exclusion assay is a widely-used technique to determine living cells (non-blue) from dead cells (blue); however, it has been reported that it overestimates cell viability and underestimates non-viable cell density [30]. In Fig. 3, at the early time point, the MTT results (c panel) and the percentage of dead cells (e panel) showed a congruent response: a decrease in MTT and an increase in the number of dead cells at the 3 min measurement. Exposure to 5 ng of mitochondria increased the MTT labeling, although it induced a slight increase in dead cells. This effect could be related to cytotoxicity. A recovery of the MTT staining, which reflects metabolic activity, was observed in the 3 min exposure conditions and repaired by PAMM MitoCeption. Cells exposed to 3 min of UVR and which were MitoCepted showed a greater decrease in the percentage of dead cells as compared with cells exposed to 3 min of UVR but which were not MitoCepted. At the early time point, even Trypan Blue differentiates living from dead cells, it does not provide information about cells that could have been weakened by UVR and MitoCeption and which could die after 18 h.

After 18 h (Fig. 3), cells continued to die and accumulated from the early time point in the conditions of 3 min UVR exposure and 3 min UVR plus MitoCeption, resulting in the increased percentage of positive Trypan Blue cells at the late time point (non-viable) (panel f). This result demonstrated that the number of dead cells increased after 18 h under the most stressful conditions. These conditions were cells exposed to 3 min of UVR. It seems that the initial Trypan Blue staining could have underestimated the number of weakened cells. The MTT conversion to formazan crystals in the cells at 18 h can only be performed by living cells under all the conditions (panel d); this reaction occurs independently of the increase in the percentage of dead cells (panel f), which did not affect the overall MTT assay result.

mRNA was isolated to measure p53 gene expression levels after the PBMCs were exposed to UVR and cultured for 2 h. Results showed that PBMCs exposed to radiation increased their p53 mRNA levels in a response that could be associated with the induction of cell arrest or repair of mtDNA [31]. The decreased recognition of mtDNA sequence by hMito primers in cells exposed to UVR and the increased p53 mRNA expression could imply that these effects are linked. However, when PBMCs were rescued by adding 2.5 ng of exogenous mitochondria, mtDNA was recovered and p53 expression levels were decreased. The mechanism by which exogenous mitochondria transfer interacts with p53 gene expression is unknown and could influence future research on how MitoCeption could inhibit damage or senescence response [31].

Our article showed that the in-vitro use of PAMM MitoCeption could repair UVR damage in our cellular model. Even if the MitoCeption technique is limited to an in-vitro AMT/T, our protocol allows an effective and proportional exogenous mitochondrial uptake in cell lines and primary cells in proportion to their dose. This technique and properties could be used for ex-vivo experimentation.

It is still a challenge to standardize and predict proportional uptake in-vivo AMT/T by damaged tissue; this is true whether exogenous mitochondria are delivered to the tissue by direct or systemic injection. Nevertheless, the therapeutic value of exogenous mitochondria for patients is supported by medical evidence [10, 16]. MitoCeption in combination with PAMM could have clinical applications in the ex-vivo transfer of mitochondria to target cells in order to repair or modify them prior to reintroducing them to a patient’s body. In the future, the MitoCeption technique could be used as part of a protocol to isolate primary cells with direct or indirect mitochondrial malfunctions, transfer/transplant exogenous mitochondria, and reintroduce them into the source organism. Today, the ex-vivo modification of cells for therapeutic purposes (such as in CAR-T cell generation and others) has been applied in anti-cancer medicine [32–36]. MitoCeption or other mitochondria transfer/transplant techniques could be used as in CAR-T cell therapy design and clinically applied to patients in future studies.

## Conclusion

In conclusion, we showed for the first time that PAMM MitoCeption can repair the decrease in metabolic activity, mitochondrial mass, and mtDNA sequence stability, decrease p53 gene expression, and decrease percentage of dead PBMCs, all of which are caused by UVR damage. We used an adaptation of the MitoCeption technique in PBMCs, which not only allows an effective and proportional uptake in primary cells, but also reduces the time of the protocol for better efficiency in downstream applications. This study opens the possibility to use mitochondria from different donors (PAMM) to treat UVR stress and possibly other metabolic diseases or malfunctions in cells in ex-vivo applications.

## Methods

### PBMCs isolation

Blood was collected from healthy female and male donors between 20 and 30 years old, each of whom provided written informed consent in accordance with relevant guidelines and regulations for mitochondria studies approved by the Bioethics Committee (the Institutional Review Board) of the Universidad San Francisco de Quito (study code: 2017-026IN). After blood collection, PBMCs were isolated using Ficoll-Paque Premium (GE Healthcare Life Sciences; MA, U.S) and centrifuged at 400 x g for 30 min without break or acceleration in order to create a density gradient. PBMCs were washed by suspension with 1X phosphate buffered saline (PBS) (Gibco by Life Technologies, ThermoFisher Scientific; MA, U.S) in a standardized volume of 10 mL and centrifuged at 1500 x g for 20 min at 4 °C. Supernatant was discarded and the pellet was re-suspended in 10 mL of PBS. Cells were counted in a Neubauer cell counting chamber and diluted to 1 × 10^6^ cell/mL in 1% FCS RPMI (Gibco by Life Technologies, ThermoFisher Scientific, MA, U.S.), resulting in a final volume of 2 mL in an Eppendorf tube; one such tube was prepared for each condition. Cells were stored for a maximum of 2 h at 4 °C until further assays were performed.

### UVR exposure

Isolated cells were incubated at room temperature for 20 min prior to UVR exposure. Cells stored at low temperature seemed to resist irradiation better, thus creating variability in the results (data not shown). PBMCs were exposed to a UVR wavelength of 254 nm (UVC) emitted by a BS-02 UVR system (Purifier Logic Class II, KS, U.S). The UVC exposure dosages were: 2 min (48 mJ/cm^2^), 3 min (72 mJ/cm^2^), 4 min (96 mJ/cm^2^), 5 min (120 mJ/cm^2^), and 6 min (144 mJ/cm^2^).

### Mitochondrial isolation

Mitochondria was isolated from 10 to 20 × 10^6^ of fresh PBMCs using the Mitochondria Isolation Kit for Tissue (ThermoFisher Scientific, MA, U.S) following manufacturer’s instructions with a final recovery step using a centrifugation at 3000 x g for 15 min to obtain highly purified mitochondria isolates. A second wash was performed using the same centrifugation conditions in order to discard residue of Reactive C from the kit. 1 mL of DMEM High Glucose (Gibco by Life Technologies, ThermoFisher Scientific, MA, U.S) without serum (DMEM pure) was added to re-suspend the mitochondrial pellet. Mitochondrial concentration was determined using a Pierce™ Coomassie Plus (Bradford) Assay Kit (ThermoFisher Scientific, MA, U.S). A typical mitochondria yield was 30 to 50 μg/mL. The mitochondrial solution was stored at 4 °C until MitoCeption of PBMCs was performed.

### Adapted MitoCeption protocol

PBMCs from all conditions were centrifuged at 500 x g at 4 °C for 5 min in 2 mL of RPMI 1% FCS in a microcentrifuge tube. The supernatant was discarded and 250 μL of the isolated mitochondrial solution in pure DMEM was added at different concentrations. 1.5 mL Eppendorf tubes were vortexed at maximum speed for 5 s with the 250 μL of mitochondria and PBMCs. A final centrifugation was performed at 500 x g for 5 min at 4 °C. Finally, cells were co-incubated at 37 °C for 1 h, washed with 1 mL of PBS, and left to rest until downstream assays were performed. PAMM is composed of the PBMCs of at least three donors (Fig. 2.1).

### Percentage of dead cells assessment

After UVR exposure, MitoCeption, and after the early and late time points, cells were washed and re-suspended in 250 μL of 1% FCS RPMI. A 10 μl aliquot of each exposed, non-exposed, treated, and un-treated cells with mitochondria was mixed through resuspension, with 10 μL of 0.02% Trypan Blue staining (Gibco by Life Technologies, ThermoFisher Scientific, MA, U.S). Two different operators counted the cells in a Neubauer cell counting chamber using an inverted Olympus microscope. Percentage of dead cells was calculated by dividing the number of dead cells (positive for Trypan Blue) by the total number of cells in the chamber.

### Mitochondria labeling with MitoTracker green®

PBMCs were incubated with 250 nm/mL of MitoTracker Green® (Molecular Probes by Life Technologies, ThermoFisher Scientific, MA, U.S) solution in 1% FCS RPMI at 37 °C during 30 min. Two washes were performed to eliminate excess dye and cells were re-suspended in 1 mL of PBS.

### Determination of the mitochondria transfer

PBMCs tested in all conditions were washed with 1 mL of PBS and centrifuged at 500 x g for 5 min prior to the assay to eliminate un-transferred mitochondria. MitoCepted PBMCs passed directly to flow cytometry analysis to determine the transfer success. Cell viability was verified (selection of cells - for Annexin and 7AAD). During the flow cytometry analysis, we focused on the lymphocyte or monocyte population using size, granularity, and CD3+ or CD14+ labeling. PBMCs were pelleted by centrifugation at 500 x g for 5 min, supernatant was discarded, and fresh RPMI + 1% FCS was added. Cells were analyzed 1 h or 18 h after MitoCeption by flow cytometry, focusing on the CD3+ lymphocyte and CD14+ monocyte populations. Cells were stained with APC mouse anti-human CD3 (555,335; BD biosciences) and PE mouse anti-human CD14 (555,398; BD Biosciences) antibodies. To determine the transfer success, a Flow Cell Cytometer Canto™ II (BD Biosciences, NJ, U.S) was used and, for the mitochondrial mass quantitation, the assays were performed using a Flow Cell Cytometer BD Accuri™ C6 Plus (BD Biosciences, NJ, U.S.). Data was analyzed by the FlowJo™, BD Accuri™ CSampler and flow cytometry Diva BD Bioscience software.

### Metabolic activity

A solution of MTT (3-(4,5-Dimethylthiazol-2-yl)-2,5-Diphenyltetrazolium Bromide) (M6494; ThermoFisher Scientific, MA, U.S) was prepared at a concentration of 20 μg/mL and filtered through a 0.45 μm filter. 100 μL of the MTT solution was added to each tested condition in 1 mL of PBMCs in 1% FCS RPMI (final MTT concentration was 200 nmol/mL), followed by a 2 h incubation. Centrifugation at 500 x g for 5 min was performed to concentrate the viable cells containing dark violet crystals. After centrifugation, we discarded RPMI plus MTT and allowed 50 μL of the medium mix to settle so as not disturb the pellet. Then, 200 μL of DMSO (sc-358,801; Santa Cruz Biotechnology, TX, U.S) was added to each condition and mixed carefully to dissolve the dark violet crystals. To perform the measurement in the microplate spectrophotometer (Epoch; BioTek Instruments), we used a 96-well ELISA plate with a flat bottom with 100 μL of each condition. The plate was read using a wavelength of 570 nm.

### Mitochondrial DNA damage quantitation by qPCR

PBMCs tested in all conditions were washed with 1 mL PBS and centrifuged at 500 x g for 5 min prior to the assay to eliminate any kind of debris or un-transferred mitochondria. DNA was extracted from PBMCs using MagMax™-96 Mutli-Sample Kit (Applied Biosystems by ThermoFisher Scientific, MA, U.S). Real time quantitative PCR was performed in a StepOne Real-Time PCR System (Applied Biosystems by ThermoFisher Scientific, MA, U.S). Fast SYBR® Green Master Mix (4,385,610; Applied Biosystems by ThermoFisher Scientific, MA, U.S) was used according to manufacturer’s instructions, resulting in a final DNA concentration of 10 ng/μL in a 10 μL reaction. mtDNA was identified and quantified using primers (hMitoF3 and hMitoR3), manufactured by Invitrogen™: hMitoF3 5′- CACTTTCCACACAGACATCA – 3′; hMitoR3 5′- TGGTTAGGCTGGTGTTAGGG – 3′ corresponding to a unique region of the mitochondria DNA between positions 241 and 390, which has been shown to be less similar to nuclear DNA sequences, thereby ensuring more accurate identification by the primers [18, 19]. The nuclear human β-2 microglobulin gene was used as a housekeeping gene with the primers: hB2MF1 5′- TGT TCC TGC TGG GTA GCT CT – 3′; and hB2MR1 5′- CCT CCA TGA TGC TGC TTA CA – 3′, as suggested by Ajaz et al.(2015) [18]. The qPCR reaction was based on the Ajaz et al. (2015) protocol with a minor modification: we included pre-incubation at 95 °C for 5 min (1 cycle); denaturation at 95 °C for 10 min; annealing and extension at 63 °C for 30 min (denaturation and extension steps were repeated for 40 cycles); and melting at 95 °C for 5 min, 63 °C for 60 min, 95 °C for 5 min, and 40 °C for 30 min. Data were collected by estimating the 2^-ΔCT^; for statistical analysis, we used the Anderson-Darling test for normality to determine the use of parametric or non-parametric analysis. After statistical normality assumptions, we used un-paired, parametric ANOVA and Student’s t-test to analyze the differences among conditions, using an alpha-value of 0.05 (*p < 0.05, **p < 0.01, ***p < 0.001, ****p < 0.0001) to establish significant differences.

### p53 gene expression

RNA was isolated from PBMCs using the PureLink™ RNA Mini Kit (12,183,025; Ambio by Life Technologies, ThermoFisher Scientific, MA, U.S). Real time quantitative PCR was performed in a StepOne Real-Time PCR System. SuperScript® III Platinum® SYBER® Green One-Step qRT-PCR Kit with Rox (11,745,500; Invitrogen by Life Technologies, ThermoFisher Scientific, MA, U.S) was used according to the manufacturer’s instructions with a final DNA concentration of 10 ng/μL in a 10 μL reaction. mtDNA was quantified using primers manufactured by Invitrogen: Tp53F 5′ - CCT CAG CAT CTT ATC CGA GTG G – 3′; Tp53R 5′ – TGG ATG GTG GTA CAG TCA GAG C – 3′; and the nuclear PUM gene was used as the housekeeping gene with the primers: PUM1F 5′- AGT GGG GGA CTA GGC GTT AG – 3; and PUM1R 5′- GTT TTC ATC ACT GTC TGC ATC C – 3′. The qPCR reaction was performed under the following conditions: pre-incubation at 95 °C for 5 min (1 cycle); denaturation at 95 °C for 15 min; annealing and extension at 60 °C for 30 min (repeat denaturation and extension steps for 45 cycles); melting at 95 °C for 15 min, 60 °C for 60 min, and 95 °C for holding. Data were collected by estimating the 2^-ΔCT^.

### ROS quantification

Aliquots of 1 million PBMCs in 2 mL of RPMI medium with 1% FCS were exposed to 6 min UVR (dosage: 72 mJ/cm^2^) in 2 mL Eppendorf tubes. After 1 h culture at 37 °C in a 5% CO2/humidified air atmosphere, exposed and non-exposed samples (controls) were MitoCepted with 2.5 ng of mitochondria, according to the method explained above. 10 μL of Dihydro-rhodamine-123 (DHR-123) (D23806; ThermoFisher Scientific, MA, U.S) was added to 400 μL of each sample (final concentration: 1 mmol/L) followed by 1 μL Phorbol 12-myristate 13-acetate (PMA) (final concentration of 1 mmol/L) (79,346; SIGMA, Merck, U.S) to induce cellular stress. Cells were cultured in the dark for 2 h. Flow cytometry was performed with a BD AccuriTM C6 Plus device (BD Biosciences, NJ, U.S) with 488 nm laser excitation and analyzed with BD AccuriTM CSampler software. Five thousand cells were collected from each sample. Results were calculated as the mean fluorescence intensity (MFI) (arbitrary units) and normalized by folding. Our data are shown with mean fluorescence intensity values.

### Statistical analyses

Data were analyzed using Minitab 17 and GraphPad Prism 6 software. The results for the mitochondrial mass and ROS production (estimated by flow cytometry), and metabolic activity (estimated with MTT assay) were normalized by using sample/control-average transformation. The percentage of dead cells was estimated using the Trypan Blue stain. The qPCR 2^-ΔCT^ fold method was applied to mtDNA quantification and expression of the p53 gene. Then, we performed an Anderson-Darling normality tests on all data sets to determine the use of parametric or non-parametric analysis. After statistical normality assumptions, we used un-paired, parametric ANOVA and Student’s t-test to analyze the differences among conditions, using an alpha-value of 0.05 (*p < 0.05, **p < 0.01, ***p < 0.001, ****p < 0.0001) to observe significant differences. Red headlines with asterisks represent the overall ANOVA significance; blue headlines with asterisks represent Student’s t-test significance between conditions and red asterisks over columns represent Student’s t-test significance between the control and the individual condition as stated in each figure.

## Additional files

Additional file 1: **Figure S1.** PBMCs lymphocytes and monocytes populations. Representative images. (PPTX 81 kb)

Additional file 2: **Figure S2.** Schematic representation of the UVR damage and PAMM MitoCeption rescue of PBMCs. (PPTX 209 kb)

## Data Availability

All data generated in this article and derived data supporting the findings of this study are available from the corresponding author upon request.

## Abbreviations

AMT/T: Artificial Mitochondrial Transfer or Transplant
CD14: Cluster of differentiation- co-receptor detection Lipopolysaccharide (LPS)
CD3: Cluster of differentiation- T-cell co-receptor
DHR-123: Dihydro-rhodamine-123
DMEM: Dulbecco’s Modified Eagle Medium
DMSO: Dimethyl sulfoxide
ELISA: Enzyme-linked immunosorbent assay
FCS: Fetal calf serum
MAV: Mean absorbance values
MFI: Mean fluorescence intensity
MSCs: Mesenchymal stem/stromal cells
mtDNA: Mitochondrial DNA
MTT: 3-(4,5-Dimethylthiazol-2-yl)-2,5-diphenyltetrazolium bromide
NADPH: Nicotinamide adenine dinucleotide phosphate
p53: Tumor suppressor gene 53
PAMM: Primary allogeneic mitochondrial mix
PBMCs: Peripheral blood mononuclear cells
PBS: Phosphate buffered saline
PMA: Phorbol 12-myristate 13-acetate
qPCR: Real-time quantitative polymerase chain reaction
RB: Respiratory burst
ROS: Reactive oxygen species
RPMI: Roswell Park Memorial Institute
UVC: UVR wavelength of 254 nm
UVR: Ultraviolet radiation
