# Supplementary material for: Primary allogeneic mitochondrial mix (PAMM) transfer/transplant by MitoCeption to address damage in PBMCs caused by ultraviolet radiation
Source: BMC Biotechnol. 2019 Jun 28;19:42. doi: 10.1186/s12896-019-0534-6 (PMC6599354; doi:10.1186/s12896-019-0534-6)
Supplement: Supplementary file 1 — Figure S1. PBMCs lymphocytes and monocytes populations. Representative images. (PPTX 81 kb) [file 12896_2019_534_MOESM1_ESM.pptx]

## Slide 1
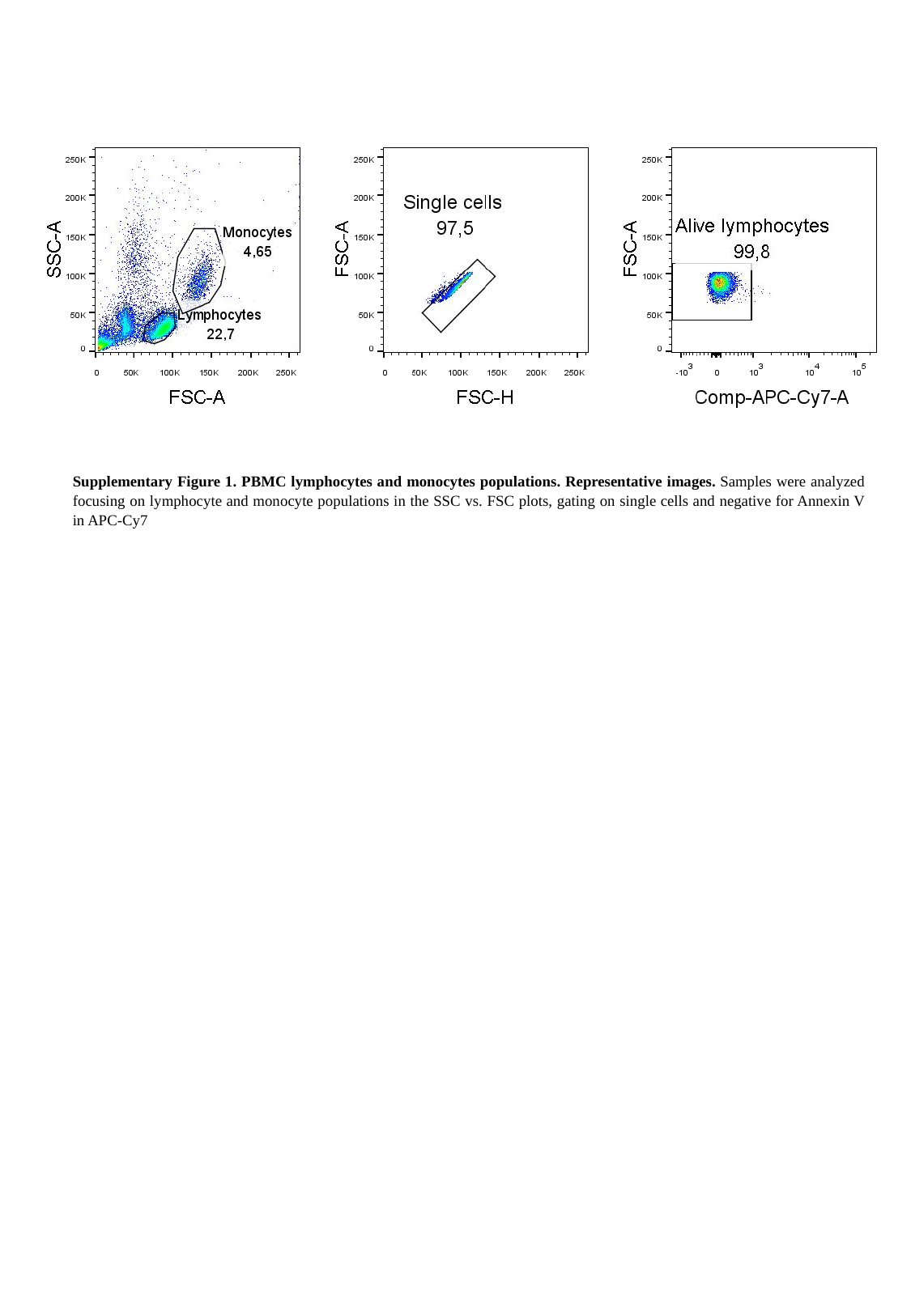

Supplementary Figure 1. PBMC lymphocytes and monocytes populations. Representative images. Samples were analyzed focusing on lymphocyte and monocyte populations in the SSC vs. FSC plots, gating on single cells and negative for Annexin V in APC-Cy7
