# Supplementary material for: Primary allogeneic mitochondrial mix (PAMM) transfer/transplant by MitoCeption to address damage in PBMCs caused by ultraviolet radiation
Source: BMC Biotechnol. 2019 Jun 28;19:42. doi: 10.1186/s12896-019-0534-6 (PMC6599354; doi:10.1186/s12896-019-0534-6)
Supplement: Supplementary file 2 — Figure S2. Schematic representation of the UVR damage and PAMM MitoCeption rescue of PBMCs. (PPTX 209 kb) [file 12896_2019_534_MOESM2_ESM.pptx]

## Slide 1
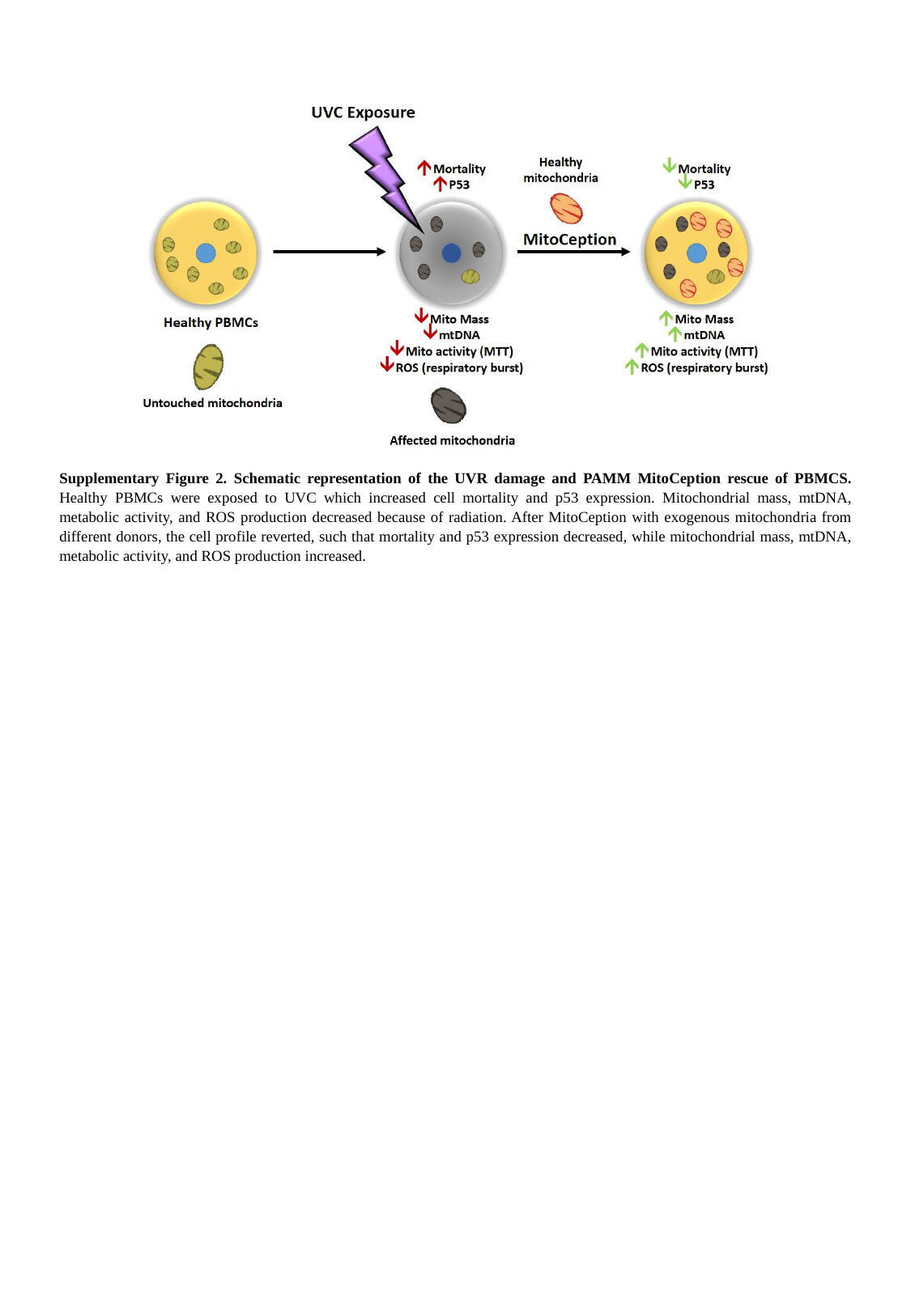

Supplementary Figure 2. Schematic representation of the UVR damage and PAMM MitoCeption rescue of PBMCS. Healthy PBMCs were exposed to UVC which increased cell mortality and p53 expression. Mitochondrial mass, mtDNA, metabolic activity, and ROS production decreased because of radiation. After MitoCeption with exogenous mitochondria from different donors, the cell profile reverted, such that mortality and p53 expression decreased, while mitochondrial mass, mtDNA, metabolic activity, and ROS production increased.
